# Comparative Analysis of Percutaneous Drainage versus Operative Drainage of Intra-Abdominal Abscesses in a Resource-Limited Setting: The Tanzanian Experience

**DOI:** 10.5334/aogh.4070

**Published:** 2023-06-01

**Authors:** Ofonime Nkechinyere Ukweh, Jared M. Alswang, Joy N. Iya-Benson, Azza Naif, Shin Mei Chan, Fabian Laage-Gaupp, Murray Asch, Vijay Ramalingam

**Affiliations:** 1 University of Calabar, Calabar, NGN; 2 Harvard Medical School, Boston, USA; 3 Muhimbili National Hospital, Dar Es Salaam, TZN; 4 Yale school of Medicine, New Haven, CT, USA; 5 Lakeridge Health, Ontario, Canada; 6 Beth Israel Deaconess Medical Centre, Boston, MA, USA

**Keywords:** Percutaneous drainage, Surgical drainage, Intra-abdominal abscess, Sub-Saharan Africa, Resource-limited setting

## Abstract

**Background:**

Intra-abdominal abscesses (IAAs) are a major cause of morbidity and mortality worldwide. While image-guided percutaneous abscess drainage (PAD) has become the standard of care in many countries, over half of the global population does not have access to interventional radiology (IR) and are left with surgery as the only option for source control.

**Objective:**

The purpose of this study is to evaluate the development, implementation, and role of a PAD service in a resource-limited setting.

**Method:**

A retrospective cohort study was performed on all patients who underwent percutaneous or surgical abscess drainage (SAD) of IAAs at Tanzania’s national referral hospital from 10/2018 to 4/2021. Patients were identified through a match case search of institutional records and inclusion was confirmed through manual chart review. Demographics, patient presentation, procedural data, and clinical outcomes were recorded in a password-encrypted database and compared between groups.

**Findings:**

Sixty-three patients underwent abscess drainage: 32 percutaneously and 31 surgically. In the PAD group, there was a 100% technical success rate and a 0% complication rate. In the SAD group, there was a 64.5% technical success rate and ten deaths within 30 days (32.3%), and one additional complication requiring major therapy (3.2%) (p < 0.001).

**Conclusion:**

Results from this study demonstrate that PAD can be performed with high technical success and without complication by trained IR physicians in Tanzania. The development of a successful PAD program exemplifies the drastic need to support the growth of IR services in this setting.

## Introduction

Global interventional radiology is a field in its infancy. From simple procedures such as core needle biopsies to more complex procedures such as transarterial chemoembolization, a well-functioning IR workforce can transform healthcare in a myriad of ways. Nowhere is the lack of IR presence more severe than in sub-Saharan Africa (SSA), where over one billion do not have access to IR services [1]. While countless applications of the specialty have the potential to make a large impact on patient care in resource-limited settings, such as SSA, data supporting their role in this context is extremely limited. As such, the adoption of the specialty in SSA is contingent on meticulous research to define its exact role in the region and ensure that growth in interventional radiology is guided by evidence.

In SSA, one such application of IR of particular interest is percutaneous drainage of intraabdominal abscess (IAA), a major cause of morbidity and mortality worldwide [2]. Prompt treatment of IAA is paramount with mortality rates reported as low as 11% when treated to as high as 80% when left untreated [2]. Previously, surgery was the only option to drain IAAs. However, because of the invasiveness of a surgical approach, its associated cost, and higher risks of morbidity and mortality, it has fallen out of favor in recent decades and is largely reserved only for cases not amenable to percutaneous drainage [3]. Conversely, image-guided percutaneous abscess drainage has become the first-line procedural management option in most clinical settings given that, when compared to surgery, it has lower risk for complications, shorter hospitalization stays, no requirement for general anesthesia, faster recovery times, and similar efficacy [3,4,5,6].

Regrettably, due to the dearth of trained IRs in SSA, the only option afforded to most in the region for the management of IAA is surgery. However, in Tanzania, this is beginning to change. In 2018, Tanzania’s first IR training program and service was initiated through international collaboration and support from the organization Road2IR. Due to there being no IRs in Tanzania prior to this, this program utilized a novel training model, where visiting teams of IR faculty and support staff participated in regular teaching trips to rapidly scale-up IR services and develop self-sustainability [1].

Percutaneous abscess drainage (PAD) has since become regularly available to patients at the nation’s largest hospital and referral center, Muhimbili National Hospital (MNH). Like many procedures offered by IR, it was hypothesized that PAD would be an effective solution to reduce the morbidity and mortality of an under-addressed cause of illness in SSA. However, there has been little reported data on implementing a percutaneous drainage program in resource-limited settings. Therefore, this study aims to assess the safety and technical feasibility of introducing PAD at a large tertiary care center in Tanzania in comparison to the regional standard of surgery.

## Materials and Methods

### Study design

A HIPAA Compliant, IRB-approved retrospective cohort study was conducted on all patients who underwent percutaneous or surgical drainage of an intraabdominal abscess between October 2018 and April 2021 at a tertiary referral center in Tanzania. Patients were identified utilizing a match case search from institutional interventional radiology and surgery records on the following words: “intraabdominal abscess”, “pelvic abscess”, and “abdominopelvic collection”. Patient records were then manually reviewed and all patients, over the age of 18, verified to have an abdominopelvic collection greater than 3cm in diameter managed by either percutaneous or surgical drainage during the study period were included in the study. Patients with perianal abscesses were excluded. This search yielded 63 patients. All cases included in the study had diagnoses confirmed by ultrasound, CT, and/or MRI. In all included patients, demographics, laboratory values, radiological findings, procedural data, and clinical outcomes were extracted from institutional records and recorded on an anonymized, password-encrypted database.

### Percutaneous drainage technique

All patients were evaluated and their imaging reviewed by a senior interventional radiologist to determine technical feasibility. Coagulopathy – International Normalized Ratio >2 and platelet count <50,000 – and no clear path for access were considered absolute contraindications. Any patient not on antibiotics was given IV ceftriaxone 1g and IV metronidazole 500mg 30 minutes prior to the procedure. Access to the abscess collection was obtained either via Seldinger or trocar methods using 18/20/21 Gauge (G) system under ultrasound or CT guidance. When the Seldinger method was used, the tract was dilated using serial tissue dilators and guidewires, most commonly an 0.035 inches Amplatz stiff wire. Following the tract dilatation, a 12 or 14 French drainage catheter was inserted. When the anatomical location was favorable, typically in more superficial abscess collections, a 12 or 14 French drainage catheter was placed by direct trocar method. Exudate was aspirated and sent for culture and sensitivity.

Patients were instructed to flush drainage catheters with 10cc normal saline at least twice daily, and keep daily output records from the catheter. Patients had follow-up imaging – ultrasound or CT – and laboratory tests, completed 72 hours post-drain placement. Drainage catheters were removed if output was consistently less than 20cc daily over three days, clinical resolution of symptoms or sepsis, and minimal collection less than 3 cm within the abscess cavity.

### Statistical analysis

Severity of presenting illness were compared between groups by level of hospitalization (ICU/ward/outpatient), volume of abscess, and WBC counts. Volume of fluid drained from the abscess served as a proxy to abscess size. Clinical outcomes were compared between groups by technical success of procedure, 30-day mortality, adverse events requiring major therapy, and presence of leukocytosis (defined as >10,000/μL) at 72-hours follow-up. Categorical data was summarized using frequencies and proportions and compared using Chi-square tests or Fisher’s Exact Test in nonparametric group comparisons. Continuous data was summarized using means and standard deviation and compared using two-sample t-tests. Descriptive and comparative analyses were calculated using IBM Statistical Package for Social Sciences (SPSS) version 22 (IBM Corp., Armonk, NY) and a p-value less than 0.05 was considered statistically significant.

## Results

### Study population

**Figure 1:**
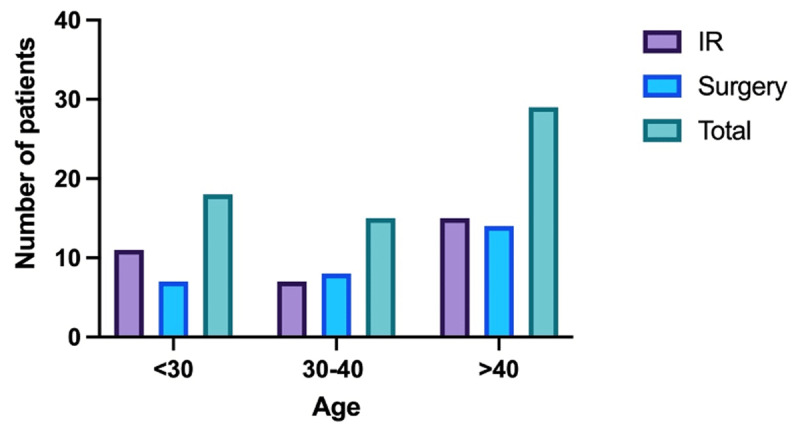
Summary of number of patients and Treatment type in different age groups.

A total of 63 patients were identified and included in the study: 32 (50.8%) underwent image-guided percutaneous abscesses drainage and 31 (49.2%) surgical abscess drainage (SAD). There was no statistically significant difference in mean age between PAD (39.5 ± 21.5 years) and SAD (42.1 ± 17.6 years) patients (p = 0.128) (Figure 1). Overall, 63.5% of patients (n = 40) were male and 36.5% female (n = 23). There was no significant difference in sex between PAD and SAD groups (p = 0.05) (Table 1). There were also no statistically significant differences in prevalence between groups for the following IAA etiologies: post-operative, traumatic, and immunocompromised (FET = 5.066; p = 0.072). The mean WBC count prior to procedure in the PAD group was 11.18 compared to 14.48 in the SAD group (p = 0.039). All SAD patients were inpatients compared to 12 PAD patients (37.5%) being outpatients (χ2 = 14.360, p < 0.001). In addition, abscess volume drained was significantly higher among SAD patients: >500cc in 93.4% (n = 29) of patients in the SAD group compared with 34.4% (n = 11) of PAD patients (χ2 = 21.823, p < 0.001) (Table 2).

**Table 1:** Demographics of the PAD and SAD groups (sex and age).


	OVERALL [N (%)]/[MEAN (SD)]	PAD [N (%)]/[MEAN (SD)]	SAD [N (%)]/[MEAN (SD)]	P-VALUE

**Sex**				

*Male*	**40 (63.5)**	**21 (65.6)**	**19 (61.3)**	**0.050**

*Female*	**23 (36.5)**	**11 (34.4)**	**12 (38.7)**	

**Age (years)**	**40.8 (19.5)**	**39.5 (21.5)**	**42.1 (17.6)**	**0.128**


**Table 2:** Baseline disease presentation of the PAD and SAD groups (abscess volume, level of care, and leukocyte count).


	OVERALL [N (%)]/[MEAN (SD)]	PAD [N (%)]/[MEAN (SD)]	SAD [N (%)]/[MEAN (SD)]	P-VALUE

**Abscess Volume (mL)**				

*<500*	24 (38.1)	21 (65.6)	3 (9.7)	<0.001

*500 – 1000*	27 (42.9)	9 (28.1)	18 (58.1)	

*>1000*	12 (19.0)	2 (6.3)	10 (32.3)	

**Level of Care**				

*Inpatient*	51 (81.0)	20 (62.5)	31 (100.0)	<0.001

*Outpatient*	12 (19.0)	12 (37.5)	0 (0.0)	

**WBC (Thousands)**	12.80 (6.37)	11.18 (6.10)	14.48 (6.30)	0.039


### Technical success and clinical outcomes

All PADs were performed with a 100% technical success rate compared to SAD, which only 64.5% (n = 20) were considered technically successful. At 72-hour follow-up, 0% of patients in the PAD group had leukocytosis (>10,000/μL) compared to 41.9% (n = 13) in the SAD group (χ2 = 16.908, p < 0.001). None of the PAD patients had major complications (SIR class C–F) following the procedure compared to surgical patients, in which 35.5% (n = 11) had a major complication (FET = 14.770, p < 0.001). The 30-day mortality rate among the SAD group was 32.3% (n = 10), while no patients died within 30 days in the PAD group 100% (χ2 = 12.270, p < 0.001) (Table 3).

**Table 3:** Clinical outcomes of patients in the PAD and SAD groups (technical success, complications, and leukocytosis).


	OVERALL [N (%)]/[MEAN (SD)]	PAD [N (%)]/[MEAN (SD)]	SAD [N (%)]/[MEAN (SD)]	P-VALUE

**Technical Success**				

***Yes***	**52 (82.5)**	**32 (100.0)**	**20 (64.5)**	**<0.001**

***No***	**11 (17.5)**	**0 (0.0)**	**11 (35.5)**	

**Complications**				

***Death***	**10 (15.9)**	**0 (0.0)**	**10 (32.3)**	**<0.001**

***Major Therapy***	**1 (1.6)**	**0 (0.0)**	**1 (3.2)**	

**Leukocytosis (72 hrs.)**				

***Yes***	**13 (20.6)**	**0 (0.0)**	**13 (41.9)**	**<0.01**

***No***	**50 (79.4)**	**32 (100.0)**	**18 (58.1)**	


## Discussion

While many strides have been made to curb the impact infectious diseases have in the region, potential contributions by certain fields in healthcare have been under-utilized, and this is especially true for interventional radiology [1,7,8]. The role of IR in resource-limited settings is still being defined, but has considerable promise to improve clinical outcomes for both non-communicable and communicable diseases alike – providing patients with options for many indications with or without surgical alternatives. In SSA, post-surgical mortality rates are twice as high as the global average [9]. This stark disparity underpins the need for more minimally-invasive approaches to manage disease and magnifies the gap that IR services are well-suited to fill in SSA. In the case of IAA, where there is already a well-established, evidence-based alternative to surgery, commonly utilized for decades around the world, the potential role of PAD in the region is clear.

However, Muhimbili National Hospital, despite being the national referral center in Tanzania, did not have capacity to perform PAD until an IR service was established at the institution in 2018. As one of the first hospitals in the region to offer this service, and limited research either supporting or refuting its role in other resource-limited settings, thorough research on the suitability of PAD within this context is of the utmost importance. Therefore, this study provides preliminary data that by offering a structured training program in IR, initiation of a percutaneous drainage program is both technically feasible and safe [1].

Source control is an essential management strategy to reduce morbidity and mortality in infectious diseases. Image-guided percutaneous drainage has been routinely offered in most high- and middle-income countries (HMICs) since its introduction in the early 1980s [10,11]. Yet nearly 40 years later, this minimally invasive option is still unavailable to the over one billion people in SSA who do not have access to IR [1]. This is especially troubling given that SSA harbors the largest burden of infectious diseases among any region [12].

In concordance with experiences reported in outside literature from HMICs, our analysis demonstrated similar benefits from managing IAA with PAD compared to SAD in Tanzania. While many studies are old, due to the longstanding establishment of outcome superiority of PAD in other geographic settings and more recent research focused on complex applications of PAD in question, reduced complication and 30-day mortality rates was similarly observed in our study [5,13,14,15]. In terms of major complications, there were zero observed in our PAD group. Complication rates were not only lower than the SAD group (34.5%), which included commonly observed complications such as wound dehiscence and fistulization [13,10] but also lower than reported for other PAD patients in outside studies (25) [5]. Thirty-day mortality rates were similarly favorable in the PAD group at 0%, lower than the 32.3% observed in the SAD group, as well as in PAD patients in outside studies [5].

However, there were inherent differences between the PAD and SAD study groups. In the SAD group, all patients were admitted as inpatients prior to surgery as compared to 62.5% in the PAD group. In addition, there was also a comparatively more elevated mean WBC count and a higher mean volume of exudate drained, suggesting larger abscess sizes. As such, worse clinical outcomes among SAD patients can at least partially be attributed to greater severity of baseline illness. While this acknowledgement is important, it does not detract from the role PAD can play in resource-limited settings, as PAD should not be considered competitive to SAD in all cases. Rather, proper patient selection of PAD is critical to optimize outcomes and avoid surgery only when evidence supports it. In certain instances, surgical drainage may be preferential and should be considered on a case-by-case basis, including but not limited to, when there is no safe pathway for drainage, multiple or multiloculated abscesses, necrotic tissue requiring debridement, and infected tumor [3]. However, the majority of patients in the SAD group in our study did not have relative contraindications to PAD and would have been suitable candidates.

There are several limitations to this study that must be considered. One such limitation is that this was a retrospective, observational study. As such, there was no randomization between groups, which introduced various confounding factors, such as illness severity as mentioned above, that may have contributed to the differences in clinical outcomes between groups. In addition, there are various shortcomings within the institution’s electronic recordkeeping infrastructure which made it a challenge to collect comprehensive baseline and follow-up data. For example, ultrasound images are not uploaded to the hospital’s picture archiving and communication system and are unavailable for retrospective review, requiring volume drained from abscess to serve as a proxy for abscess size in this study. In addition, the surgical and interventional radiology units utilize a different system for charting. In addition to the hospital’s electronic medical record (EMR), the IR division also uses a supplementary Research Electronic Data Capture (REDCap) database for both clinical and research purposes. The REDCap database is generally more detailed than the EMR, which may have accounted for some differences between groups.

Improving access to interventional radiology services and procedures in resource-limited settings needs to be prioritized in global health. Even introducing relatively simple, but essential non-vascular procedures, can have tremendous impact on quality of life and can even be life-saving. Such procedures, including nephrostomy tubes, biliary drains, and core needle biopsies, in Tanzania have already been shown to be technically feasible, safe, and produce clinical outcomes on par with international standards [16,17,18]. A structured, locally-based IR training program has shown to be an effective means to accomplish this [1] and provided the context in which this study took place. Percutaneous abscess drainage is in this category of procedures, providing immense benefits for patients in terms of reduced mortality, complication rates, length of hospitalization, and costs incurred [5,10]. Results from this study mirror the positive impact of PAD observed in HMICs and signifies that, through formalized training in IR, a PAD program can be effectively developed in a resource-limited setting to improve outcomes in patients with IAA.
